# Variations in competencies needed to complete surgical training

**DOI:** 10.1002/bjs5.50200

**Published:** 2019-08-01

**Authors:** S. Wood, O. P. James, L. Hopkins, R. Harries, D. B. T. Robinson, C. M. Brown, T. Abdelrahman, R. J. Egan, W. G. Lewis

**Affiliations:** ^1^ Wales Deanery PGMDE School of Surgery Health Education and Improvement Wales, Cefn Coed Nantgarw UK; ^2^ Department of Surgery Morriston Hospital, Heol Maes Eglwys Swansea UK

## Abstract

**Background:**

This study aimed to analyse the degree of relative variation in specialty‐specific competencies required for certification of completion of training (CCT) by the UK Joint Committee on Surgical Training.

**Methods:**

Regulatory body guidance relating to operative and non‐operative surgical skill competencies required for CCT were analysed and compared.

**Results:**

Wide interspecialty variation was demonstrated in the required minimum number of logbook cases (median 1201 (range 60–2100)), indexed operations (13 (5–55)), procedure‐based assessments (18 (7–60)), publications (2 (0–4)), communications to learned associations (0 (0–6)) and audits (4 (1–6)). Mandatory courses across multiple specialties included: Training the Trainers (10 of 10 specialties), Advanced Trauma Life Support (6 of 10), Good Clinical Practice (9 of 10) and Research Methodologies (8 of 10), although no common accord was evident.

**Discussion:**

Certification guidelines for completion of surgical training were inconsistent, with metrics related to minimum operative caseload and academic reach having wide variation.

## Graphical abstract

Certification guidelines for completion of surgical training are inconsistent and demonstrate significant interspecialty variance. Consensus regarding the competencies required for independent surgical practice is required urgently.

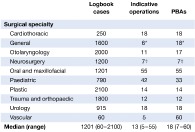

Curricular harmonization needed

## Abstract (es)

**Antecedentes:**

Este estudio se propuso analizar el grado de variación relativa en las competencias específicas de la especialidad que se requieren para obtener el certificado de haber completado la formación (*Certification of Completion of Training*, CCT) por el *Joint Committee for Surgical Training* (JCST) del Reino Unido.

**Métodos:**

Se analizaron y compararon las guías del organismo regulador relacionadas con las competencias en habilidades quirúrgicas, tanto operatorias como no operatorias, requeridas para el CCT.

**Resultados:**

Se demostró una amplia variación entre especialidades en el número mínimo requerido del cuaderno de casos (mediana 1.201; rango 60‐2.100), operaciones índices (13; 5‐55), evaluaciones basadas en procedimientos (18; 7‐60), publicaciones (2; 0‐4), comunicaciones para determinar asociaciones (0; 0‐6) y auditorias (4; 1‐6). Los cursos obligatorios entre las distintas especialidades incluían: formación de los formadores (10 de 10 especialidades), apoyo vital avanzado en traumas (6/10), buena práctica clínica (9/10) y metodologías clínicas (8/10), aunque era evidente que no existía un acuerdo común.

**Conclusión:**

Las directrices sobre la certificación para completar la formación quirúrgica eran inconsistentes, con una amplia variación en los números relativos a los mínimos casos operados y objetivos académicos alcanzados.

## Introduction

Surgical training has historically been by apprenticeship, relying on clinical exposure, although more recently the UK training model has become more competency‐based, following introduction of the Intercollegiate Surgical Curriculum Programme platform, incorporating work‐based assessments and logbook case evaluation. From an international perspective, training certification requirements differ from those in the UK^1^, ^2^. Relatively few countries have embraced competency‐based curricula; for example, only five of 11 general surgery training programmes stipulate academic, management and operative competencies^2^.

In most of these healthcare systems, curricula are scrutinized regularly with repetitive appraisal and modification. Modern curricula contain several components: explicit (taught subjects and competencies in a defined mission); implicit (lessons arising from school culture); hidden ( ethical, moral or value‐based lessons learnt without explicit intention); excluded; and extracurricular^3^.

Despite an imperative to standardize competency‐based training, anecdotally there appears to be wide disparity among surgical specialties regarding the competencies required for certification of completion of training (CCT). Ten individual surgical specialties are recognized by the UK Joint Committee on Surgical Training (JCST): cardiothoracic surgery, general surgery, otolaryngology, neurosurgery, oral and maxillofacial surgery (OMFS), paediatric surgery, plastic surgery, trauma and orthopaedic surgery, urology, and vascular surgery^4^. In addition, general surgery trainees must nominate their choice from eight specialist interests: breast, colorectal, upper gastrointestinal, vascular, transplantation, endocrine, general surgery of childhood, and advanced trauma^5^. The aim of this study was to analyse the extent of variation in the specialty‐specific competencies required for CCT by the JCST across surgical specialties.

## Methods

Certification guidelines for all ten surgical specialties (2017–2018 updates) were obtained via the JCST website^4^. These guidelines for CCT are produced by each of the ten Specialty Advisory Committees (SACs) and published under the auspices of their parent body, the JCST. Each guideline incorporates the same broad aspects and domains, including objective measures such as: minimum number of operative logbook cases, work‐based assessments including clinical case‐based discussions and procedure‐based assessments (PBAs) at a denoted competence level; peer‐reviewed publications; communications to learned societies; audits; and continuing professional development by means of mandatory courses. These documents were analysed in a quantitative and qualitative manner, and the findings compared between specialties. When the minimum number of PBAs was not specified, it was determined as the need to demonstrate competence at a given level on a single occasion for each indexed operation in that specialty.

For general surgery, there are two distinct elements to certification requirements: those relating to elective and emergency general surgery, and those relating to a trainee's chosen specialist interest. For the purposes of analysis, both components were analysed independently.

### Statistical analysis

Statistical analysis appropriate for non‐parametric data was performed using SPSS® version 25 (IBM, Armonk, New York, USA). Bivariable correlation was calculated with Spearman's ρ (non‐parametric), with statistical significance set at *P* < 0·050.

## Results

Complete certification guidelines were identified for the ten specialties: cardiothoracic surgery, general surgery, otolaryngology, neurosurgery, OMFS, paediatric surgery, plastic surgery, trauma and orthopaedic surgery, urology, and vascular surgery.

Minimum operative caseload, number of indicative operations and the minimum number of PBAs required in indexed operations are summarized in *Table* 1. A wide variety of logbook cases were required, ranging from 60 in vascular surgery to 2100 in plastic surgery (median 1201). Similarly, vascular surgery had the lowest number of indexed operations (5), compared with 55 for OMFS (median 13). Conversely, vascular surgery required the largest number of PBAs (60), compared with a minimum of 7 for neurosurgery (median 18). It should be noted that neurosurgical indexed operations were classified into seven groups; thus, a minimum of seven indexed operations may be performed (1 per group), but could include up to 87 indexed operations in total.

**Table 1:** Summary of mandatory operative experience, indicative operations and procedure‐based assessments related to specialty

	Logbook cases	Indicative operations	PBAs
**Surgical specialty**			
Cardiothoracic	250	18	18
General	1600	6*	18*
Otolaryngology	2000	11	17
Neurosurgery	1200	7†	7†
Oral and maxillofacial	1201	55	55
Paediatric	790	42	33
Plastic	2100	14	14
Trauma and orthopaedic	1800	12	12
Urology	915	18	18
Vascular	60	5	60
**Median (range)**	1201 (60–2100)	13 (5–55)	18 (7–60)

* General surgery trainees have six indexed general surgery procedures, but must also demonstrate competence in additional procedures according to their stated specialist interest; see *Table* 2.

† Neurosurgery indexed procedures are divided into seven groups; thus, in theory, trainees need demonstrate competence in a minimum of only seven procedures. PBA, procedure‐based assessment.

There were distinct variations in specialist interest requirements for general surgery trainees (*Table* 2). Emerging or smaller subspecialties (transplantation, endocrine surgery, general surgery of childhood, advanced trauma) were unable to quote minimum operative caseload requirements due to fewer trainees. No significant interspecialty correlation was found between indicative surgical procedures or PBAs.

**Table 2:** Additional competency requirements by specialist interest for general surgery trainees

	Indicative operations	PBAs
**Specialist interest**		
Breast	4	27
Colorectal	5	18
Upper gastrointestinal	4	21
Vascular	4	15
Transplant	n.a.	12
Endocrine	n.a.	9
General surgery of childhood	n.a.	9
Advanced trauma	n.a.	18
**Median (range)**	4 (4–5)	17 (9–27)

Indicative operations requiring a minimum operative caseload differ to those operations requiring procedure‐based assessments (PBAs) according to certification guidelines^6^. n.a., Not applicable.

Minimum academic requirements are shown in *Table* 3, outlining the absolute minimum number of publications, communications to learned societies and audits required for CCT. In OMFS, plastic surgery, and trauma and orthopaedic surgery, publications and/or presentations may be included as part of the minimum requirements but could technically be substituted with predetermined equivalents, including higher degrees, and patient recruitment into research projects. Publication requirements ranged from zero (OMFS, trauma and orthopaedic surgery) to four (cardiothoracic surgery, paediatric surgery). Similarly, national presentation requirements range from zero in six of ten specialties to six in cardiothoracic surgery. Audit requirements were also variable, ranging from 1 to 6 per training programme (median 4).

**Table 3:** Summary of academic requirements for certification related to specialty

		Presentations*		
	Publications	Regional	National	Audits
**Surgical specialty**				
Cardiothoracic	4	n.a.	6	1
General	3	3	0	3
Otolaryngology	2	0	0	6
Neurosurgery	2	n.a.	2	1
Oral and maxillofacial	0†	0†	0†	5
Paediatric	4	n.a.	4	6
Plastic	2	0†	0†	6
Trauma and orthopaedic	0†	0†	0†	6
Urology	2	2	0	3
Vascular	3	n.a.	2	3
**Median (range)**	2 (0–4)	0 (0–3)	0 (0–6)	4 (1–6)

* Number required is equal to or more than the number specified.

† Publications and/or presentations may be included as part of the minimum requirements, but technically could be substituted with predetermined equivalents.

Completion of the Good Clinical Practice course was cited as desirable for plastic surgery certification, but mandatory for all other specialties. Similarly, the Research Methodologies course was considered desirable for plastic and general surgery, but mandatory for all other specialties. All specialties mandated completion of the Training the Trainers course or equivalent. Leadership and management competencies were typically demonstrated by formal completion of a course in health service management, although certain specialties (plastic surgery, trauma and orthopaedic surgery, otolaryngology) required only documented evidence of activity in that field. Some specialties required specialty‐specific courses, for example a British Association of Urological Surgeons' urodynamics course for urology. Advanced Trauma Life Support was the most common clinical cross‐specialty course, required in general surgery, neurosurgery, OMFS, plastic surgery, trauma and orthopaedic surgery, and vascular surgery. With the exception of general surgery, neurosurgery, and trauma and orthopaedic surgery, minimum attendance of 70 per cent at regional teaching was considered mandatory.

A statistically significant correlation was found between the number of national presentations required and both publications (ρ = 0·743, *P* = 0·014) and logbook caseload (ρ = −0·775, *P* = 0·009). No other statistically significant correlations were found between the clinical and academic requirements outlined in *Tables* 1 and 3 respectively (*Table* 4).

**Table 4:** Correlation between clinical and academic certification of completion of training requirements related to surgical specialty

		Presentations*					
	Publications	Regional	National	Audits	Logbook cases	Indicative operations	PBAs
**Publications**							
ρ	1·000	0·117	0·743†	−0·325	−0·606	−0·108	0·384
*P*		0·747	0·014†	0·360	0·063	0·767	0·274
**Presentations** *							
Regional							
ρ	0·117	1·000	−0·391	−0·272	0·026	−0·178	0·088
*P*	0·747		0·263	0·447	0·943	0·623	0·810
National							
ρ	0·743†	−0·391	1·000	−0·453	−0·775‡	0·058	0·236
*P*	0·014†	0·263		0·189	0·009‡	0·873	0·512
**Audits**							
ρ	−0·325	−0·272	−0·453	1·000	0·598	0·245	−0·071
*P*	0·360	0·447	0·189		0·068	0·494	0·846
**Logbook cases**							
ρ	−0·606	0·026	−0·775‡	0·598	1·000	−0·055	−0·607
*P*	0·063	0·943	0·009‡	0·068		0·881	0·063
**Indicative operations**							
ρ	−0·108	−0·178	0·058	0·245	−0·055	1·000	0·228
*P*	0·767	0·623	0·873	0·494	0·881		0·527
**PBAs**							
ρ	0·384	0·088	0·236	−0·071	−0·607	0·228	1·000
*P*	0·274	0·810	0·512	0·846	0·063	0·527	

* Number required is equal to or more than the number specified.

† Correlation significant at *P* < 0·050 level (2‐tailed);

‡ correlation significant at *P* < 0·010 level (2‐tailed). PBA, procedure‐based assessment.

## Discussion

This study has described and compared competencies required for certification between surgical specialty curricula in a national surgical training system. It has exposed wide variation in competencies required to satisfy the ten surgical specialty curricula. Individual SAC views and published opinions seemed to reflect different perceptions about the elements that determine competence.

With regard to clinical domains, minimum operative caseload requirements differed 35‐fold, numbers of indicative operations differed 11‐fold, and procedure‐based assessment requirements differed by more than eightfold between specialties. Demonstration of academic performance by peer‐reviewed article publication differed by over fourfold, whereas communications to professional associations and audits performed both differed by more than sixfold. In contrast, requirements for non‐technical skills and continuing professional development, including educational course attendance or teaching skills, were similar between specialties.

Given that all surgical specialty training in the UK falls under a single umbrella organization, the JCST, there appears to be remarkable inconsistency between specialties in the competencies required for specialist accreditation. Arguably, this is not surprising because each SAC is responsible for its own particular standard setting, with no common process of achievement. Requirements may be based on a quasiquantitative model, as in general surgery, where the indicative number threshold was based on lower quartile figures achieved by a prior (2012) trainee cohort. Even when defined criteria were used to stipulate requirements, discrepancies may occur between the requirements for certification and competence. With regard to general surgery, for example, the number of indexed procedures required for CCT has been shown previously^6^ to correspond poorly with the number of procedures required to gain competence for independent practice as assessed by PBAs.

This variation in surgical training competencies is also evident from an international perspective. General surgery operative logbook requirements range from no specified minimum in Canada, India and Italy, to 400 abdominal operations in Germany, 400–750 in Greece, and 850 in the USA^1^, ^7^. There is an expectation and presumption that surgeons exiting training and entering independent practice are trained to a common, minimum and competent standard, yet current UK and international competency requirements do not reflect this. For operative competency, some variation between specialties should be expected because of the nature and complexity of the procedures involved. However, a 35‐fold difference in logbook requirements appears extreme, and inconsistent with the principles of a competency‐based curriculum. Curricula should specify whether the goal is to reach independent competence or simply demonstrate exposure to specific procedures. Focusing on competence in a select core group of procedures, and setting specific recommendations by specialty interest, as seen in general surgery, may be preferable. Even if learning curves are consistently steep (equating to easy and rapid learning), it is arguably unrealistic to expect trainees to prove competence for independent practice in as many as 55 indicative procedures, as suggested in the OMFS curriculum. In specialties where the number of PBAs required is equal to (for example OMFS, trauma and orthopaedic surgery) or less than (paediatric surgery) the number of index procedures, competence is demonstrated by a single summative assessment by a solitary trainer. This is at odds with the educational paradigm of demonstrating incremental progression in proficiency.

Other examples of curriculum inconsistency were identified. For example, within the subspecialty of endocrine surgery, there were defined index procedures with clearly defined competency indicators (3 level 4 PBAs, by three assessors), yet no consensus existed regarding the minimum caseload required for CCT^5^.

Academic and non‐operative competencies should be less susceptible to specialty differences. A UK trainee‐led study in 2016 made panspecialty recommendations with regard to academic CCT requirements, but these have not been adopted or implemented widely^8^. Negotiating universal accord regarding minimum operative and non‐operative competencies should not be beyond the abilities of international regulatory authorities overseeing training in countries with comparable healthcare standards.

Academic performance is widely considered integral to surgical training and career progression. The benchmark used to judge such performance frequently defaults to peer‐reviewed scientific publication quantity and quality, yet the process by which peer‐reviewed publications are achieved is well recognized to be flawed^9^, ^10^, ^11^. Other metrics, such as the award of a higher degree, were historically deemed almost essential for promotion and career progression in general surgery. In 2013, achievement in this arena was embedded in the JCST curriculum in general surgery, which mandated that all trainees must be in possession of three peer‐reviewed scientific publications and have delivered three communications to learned societies to qualify for CCT^5^. Thomas and colleagues^12^ reported in 2015 that such academic criteria were met by successful applicants for CCT in General Surgery (2012–2013) for publications and presentations by 88 and 94 per cent respectively. In addition, 53 per cent of trainees had achieved a Doctorate and 22 per cent a Master's degree.

This study has limitations. Surgical specialties inherently differ and, in light of the current drive and emphasis towards competence‐based training, caseloads and numbers estimated to reach competency are unlikely to be similar. Simple numerical variations may not be that important, and it is noted that no attempt was made to categorize logbook numbers by degree of operative complexity. It is, nevertheless, one thing to be judged as being competent in a small number of simple procedures, but it takes greater experience to learn advanced operative techniques and strategies for managing complex problems. For this reason, the JCST included indicative numbers in its guidelines for CCT, and these have now been incorporated into all surgical specialty curricula, based on the level thought to be needed to deal with the full spectrum of clinical presentations and operative pathologies likely to be met in independent practice. These numbers reflected statistical analysis of the first quartile achieved by previous trainee cohorts, in some but not all specialties.

Competency‐based curricula represent positive steps forward in surgical training, but should be applied consistently. If training and clinical experience are to be optimized and consistent with the needs of the future workforce, further detailed profiling will be required, so that the transferable aspects of surgical curricula can be configured for universal purpose. Comparative perspectives related to operative skills will likely vary, but those related to non‐operative technical skills and academic reach should align.

## Disclosure

The authors declare no conflict of interest.
